# A war on many fronts: cross disciplinary approaches for novel cancer treatment strategies

**DOI:** 10.3389/fgene.2024.1383676

**Published:** 2024-05-30

**Authors:** Adriana Del Pino Herrera, Meghan C. Ferrall-Fairbanks

**Affiliations:** ^1^ J. Crayton Pruitt Family Department of Biomedical Engineering, University of Florida, Gainesville, FL, United States; ^2^ University of Florida Health Cancer Center, University of Florida, Gainesville, FL, United States

**Keywords:** mathematical modeling, cancer, ecology, evolution, economics, engineering, control theory

## Abstract

Cancer is a disease characterized by uncontrolled cellular growth where cancer cells take advantage of surrounding cellular populations to obtain resources and promote invasion. Carcinomas are the most common type of cancer accounting for almost 90% of cancer cases. One of the major subtypes of carcinomas are adenocarcinomas, which originate from glandular cells that line certain internal organs. Cancers such as breast, prostate, lung, pancreas, colon, esophageal, kidney are often adenocarcinomas. Current treatment strategies include surgery, chemotherapy, radiation, targeted therapy, and more recently immunotherapy. However, patients with adenocarcinomas often develop resistance or recur after the first line of treatment. Understanding how networks of tumor cells interact with each other and the tumor microenvironment is crucial to avoid recurrence, resistance, and high-dose therapy toxicities. In this review, we explore how mathematical modeling tools from different disciplines can aid in the development of effective and personalized cancer treatment strategies. Here, we describe how concepts from the disciplines of ecology and evolution, economics, and control engineering have been applied to mathematically model cancer dynamics and enhance treatment strategies.

## 1 Introduction

Cancer can be classified into multiple categories depending on the tissue that it originates from, the location and the organ it affects. In 2023, one in 260 children and adolescents were diagnosed with cancer before the age of 20, whereas one in three people will be diagnosed with cancer in their lifetime. Adenocarcinomas originate on mucus membranes of glandular tissues and are often classified based on the organ where they originate such as the lung, prostate, pancreas, esophagus, colon, breast, stomach, and kidney and predominantly affect adults. Additionally, adenocarcinomas account for 80%–95% of cancers in their affected organ (Maurie Markman, 2022). According to the Surveillance, Epidemiology, and End Results (SEER) Program from the National Cancer Institute, adenocarcinomas prognosis varies across types; cancers such prostate, breast, and colorectal have a 90% 5-year survival rate (Siegel et al., 2023), while other adenocarcinomas such as pancreatic, lung, esophageal, and stomach have a 5-year survival rate ranging from 35% for stomach adenocarcinomas to as low as 12% for pancreatic adenocarcinomas (Siegel et al., 2023). While the origin of these cancers is still unknown, factors such as smoking, toxin exposure, and radiation therapy are factors that contribute to the development of these solid tumors. The physical manifestations of adenocarcinoma tumors include localized (on the organ it originates from) and systemic symptoms. Localized disease can exert pressure in nearby organs and manifest as shortness of breath and coughing for lung adenocarcinomas (Blandin Knight et al., 2017), or as heartburn and regurgitation for esophageal adenocarcinomas (Sharma, 2022). Once the disease metastasizes, the symptoms can affect the entire body causing weight loss, weakness, or loss of appetite. These symptoms can indicate the need for a detailed medical examination, which may include extensive laboratory tests, imaging studies, and biopsies. Data from these tests, such as tumor volume or sequencing data, can be leveraged to inform treatment decisions (Mullangi and Lekkala, 2024). Patients diagnosed with adenocarcinomas receive different types of treatments depending on the stage and localization of the cancer, but first-line therapeutics often include a combination of surgery, chemotherapeutics, and radiation (Adenocarcinoma Cancers, 2021). Due to the heterogeneity of the disease, treatment response is highly variable motivating researchers to develop and use *in silico* tools to make informed treatment decisions.

In this review, we explore mathematical modeling frameworks from different disciplines that have been applied to model cancer progression and develop novel treatment strategies. Mathematical oncology has emerged as a discipline that aims to study cancer using mathematics often focused on developing personalized treatments using patient data (Rockne et al., 2019). Although replicating all tumor cellular interactions experimentally and relating to treatment response remains challenging, mathematical models can offer accurate simulated predictions of disease progression based on patient-specific information. We have focused on reviewing the mathematical modeling frameworks used in three different disciplines: ecology and evolutions, economics, and control engineering that have been applied to open questions in cancer research. These frameworks have been adapted to leverage a variety of different types of biological information that can be obtained from adenocarcinoma solid tumors after surgery (e.g., sequencing, histology, biomarker prevalence), through imaging tests (e.g., X-rays, computed tomography (CT), magnetic resonance imaging (MRI)) or laboratory tests (e.g., biomarker levels, cell counts). The use of mathematical models in combination with this data can provide novel insights into treatment strategies, for example, this has been shown effective such in dosing of abiraterone in metastatic prostate cancer (NCT02415621, NCT03511196). While some of these transdisciplinary applications are in their infancy, cancer researchers have provided proof of concept that adaptation of these frameworks can provide novel insights into cancer progression and treatment.

Pulling from the field of ecology and evolution, the invasive and cooperative nature of cancer cell populations have many parallels with the behavior of interacting biological populations. In nature, animal populations interact in ecosystems where they try to survive through competition or cooperation. They must also adjust to the amount of resources available and other selective pressures of their environment. Interactions between species have been widely studied to understand concepts such as adaptation, competitive exclusion, and predator-prey dynamics. Ecological population-based modeling uses mechanistic mathematical models to simulate the dynamics of different species within an ecosystem. These ecological concepts can be translated to cancer, where the animal populations become specific cancer cell populations that interact in the tumor microenvironment to compete for resources (e.g., oxygen, nutrients) and survive the intense selective pressure of treatment. Concepts from ecology and evolution have been widely applied to cancer progression and treatment response. Prey-predator interactions, also known as Lotka-Volterra, have been able to unravel interactions between cellular populations and under the effects of treatment in cancers such as prostate, lung, and breast cancer (Silva et al., 2012; Chen et al., 2019; Cerasuolo et al., 2020). Additionally, geospatial metrics from ecology have been applied to understand the spatial-temporal distribution of cancer cells in breast, lung, and colorectal adenocarcinomas using single-cell sequencing and DNA methylation data (González-García et al., 2002; Casasent et al., 2018; Dietz et al., 2019). Phylogenetic analysis is a common technique to understand evolution of different species. In cancer, phylogenetics has uncovered evolutionary trajectories of tumor populations and stratify subtypes within a tumor. This type of analysis has been applied mostly to protein-coding sequencing data of an individual obtained through whole exome sequencing (WES) in adenocarcinomas from breast, lung, esophageal and colorectal tumors (Kostadinov et al., 2013; Leung et al., 2017; Fimereli et al., 2022; Frankell et al., 2023). In this review, we explored phylogenetic tree reconstruction through a variety of techniques, namely, Bayesian inference models, maximum parsimony algorithms, and Markov chain Monte Carlo methods (MCMC). The characterization of tumor evolutionary trajectories using these approaches has the potential to aid in tumor classification and treatment selection. While ecology and evolutionary concepts offer clear translatability to cancer, leveraging tools from other mathematical modeling disciplines can further enhance our understanding of this disease.

Historically, a tool from economics known as game theory has been applied to evolutionary questions and more recently to open questions in cancer research. This mathematical framework can analyze strategic interactions between cancer populations and the clinicians’ treatment choices. When applying a game theoretic approach, different concepts would need to be defined including the game, the players, the strategies, and trade-offs or costs associate with each strategy. In cancer research, cellular interactions or treatment strategies can be considered as games, where cancer cells are the players. This theory has been broadly applied in two ways: (1) where cell populations are players that interact with each other or (2) where clinician and the tumor interact with each other through strategies decided by the former. In the context of treatment, chemotherapies, radiation therapy, or immunotherapy would act as different strategies in the games and each of them has a costs and benefits associated with it (Archetti, 2021). Whenever game theory is applied to model the interactions of cells in the cancer microenvironment or phenotypic plasticity it is also knows as evolutionary game theory (Kaznatcheev et al., 2015). In this approach, sensitive and resistant cell populations are often considered as players and treatments as strategies. Game theory has been applied to study the dynamics of cancers including non-small cell lung cancer and ductal adenocarcinoma as well as malignant cellular processes of cancer such as epithelial mesenchymal transition or spatially structured tumors (Kaznatcheev et al., 2015; Malekian et al., 2016; Nam et al., 2021). So far, the interactions of cells in a population have been modeled as independent and are only based on predetermined rules or strategies. Additional complexity is captured by the application of Stackelberg evolutionary game (SEG) frameworks, where the action of a player depends on a previous action or strategy. In SEG, the clinician will make the decision first, which is often a treatment strategy, followed by the other players in the game. The clinician’s treatment decisions can influence the dynamics and evolution of the cancer population they aim to treat, while the cancer population(s) will have to follow the laws of natural selection, including behaviors such as mutation. SEG is commonly described as a leader-follower game where the physician is the leader that can steer cancer evolution, while cancer cells are natural followers that are influenced by the treatment decisions made by the leader (Wölfl et al., 2022; Stein et al., 2023).

Finally, concepts from control engineering and control theory have been applied to study cancer progression. In this scenario, cancer cells are influenced by an external agent, in this case treatment, with the goal of reach an optimal state or tumor size by developing strategies to control tumor growth. Tumors under treatment can be viewed as a control system, where the state of the system is defined by the number of cancer cells and the control is usually the drug effect on the cells (Lecca, 2020). Control theory has been applied to study treatment strategies for lung adenocarcinoma to reach an optimal state of minimal tumor population (Jonsson et al., 2017). Additionally, these approaches have been combined with gene regulatory networks in pancreatic cancer to identify genes and molecules that can serve as control agents to drive the cancer to a desired state (Plaugher and Murrugarra, 2021).

**FIGURE 1:**
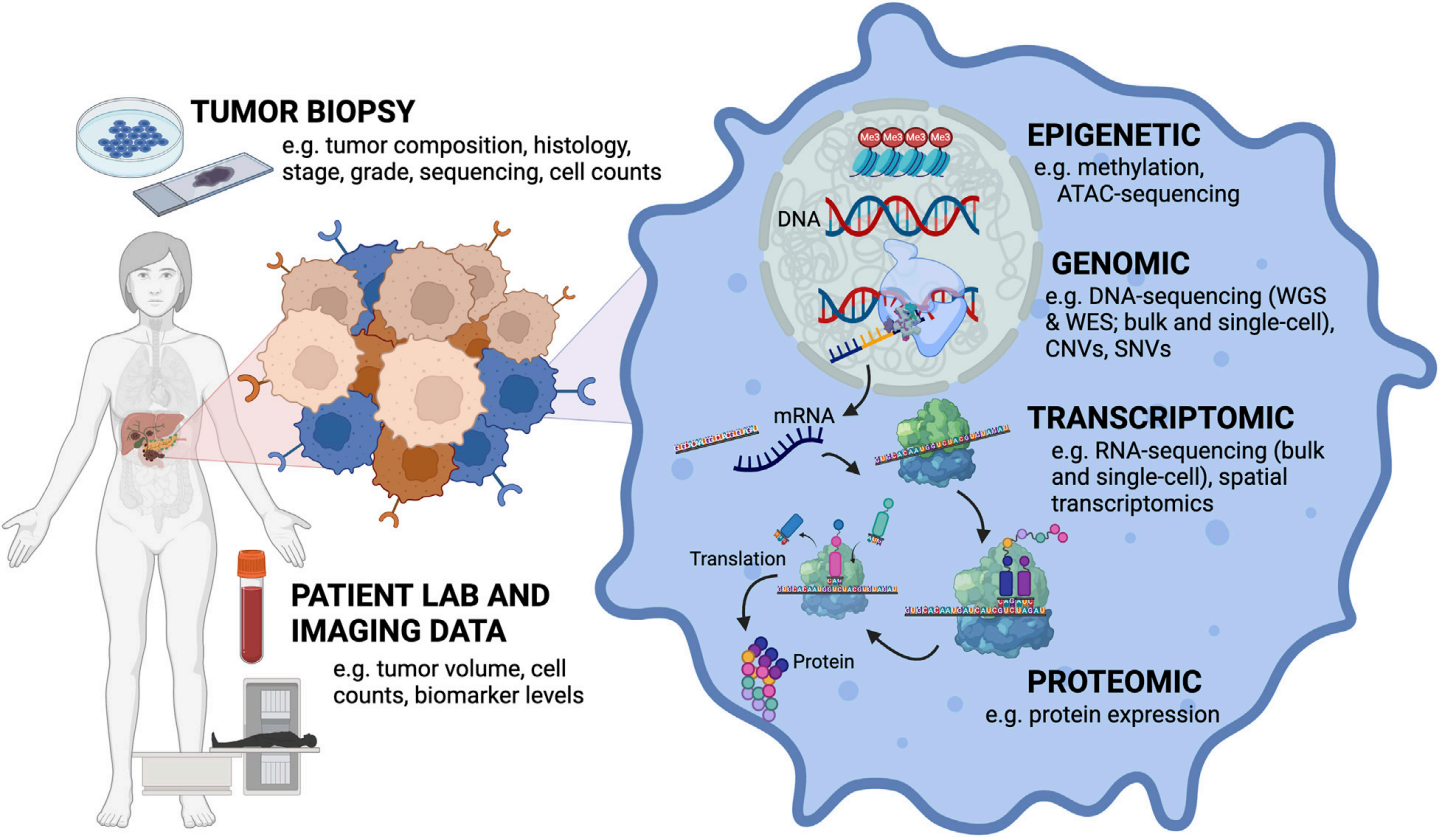
Mathematical modeling of cancer research must bridge the multi-scale nature of the different types of data that can be collected from a patient’s tumor. Input data types ranging from the tumor level to the cellular level. Tumor biopsies can provide information about tumor composition, histology, stage, grade, and sequencing can be performed on these samples to get at mutation and clonal information. Primary tumor cells can also be isolated and cultured *in vitro* to obtain cell counts. Patient lab values and imaging can collect information about tumor volume, cell counts and biomarker levels. At the cellular level, different aspects of cellular (dys)function can be assayed dependent on the type of data collected (e.g., DNA, RNA, protein, epigenetic).

Leveraging transdisciplinary mathematical modeling approaches from ecology and evolution, economics, and control engineering, cancer researchers have started to gain novel insights into cancer dynamics and create new treatment strategies. Cancer research offers a rich data source across multiple scales (Figure 1) that can benefit greatly from harnessing mechanistic cross-disciplinary insights to better understand tumor progression and treatment response. Here, we discuss how mathematical modeling tools from these different fields have been applied to understand adenocarcinoma progression and treatment response and discuss the opportunities these tools present to advancing cancer research.

## 2 Pushing beyond computational systems biology

Systems biology originates as a methodology to comprehend complex biological systems, offering a means to streamline the analysis and interpretation of multi-layer data. Systems biology focuses on the interactome or network behavior of the species of interest and their effects on the overall biological system as opposed to a reductionist biology view that will focus on a single protein or gene at a time and how it changes in response to a stimulus. An overarching goal of cancer systems biology is to develop new treatment strategies, refine drug administration schedules, and identify diagnostic biomarkers. Cancer progression can be understood as a biological system that has been studied using both experimental and computational approaches (Yalcin et al., 2020). Experimentally, cancer systems biologists leverage multi-omics data sources including genomics, transcriptomics, proteomics, metabolomics, and epigenomics to provide the comprehensive overview of the cellular differences between different samples of interest (often pre and post treatment). These data-rich sources require computational tools to get a clear picture of what is happening biologically. This is where mathematical modeling can help provide insight into disease progression and treatment response. Mathematical oncology originates from a need to characterize the interaction among cellular populations present in the tumor microenvironment. Many researchers have traditionally focused on developing mathematical models rooted in ecology and evolution frameworks, however there is an opportunity to apply mechanistic mathematical modeling approaches from a broader spectrum of disciplines to understand the dynamics of cell populations in cancer.

**FIGURE 2:**
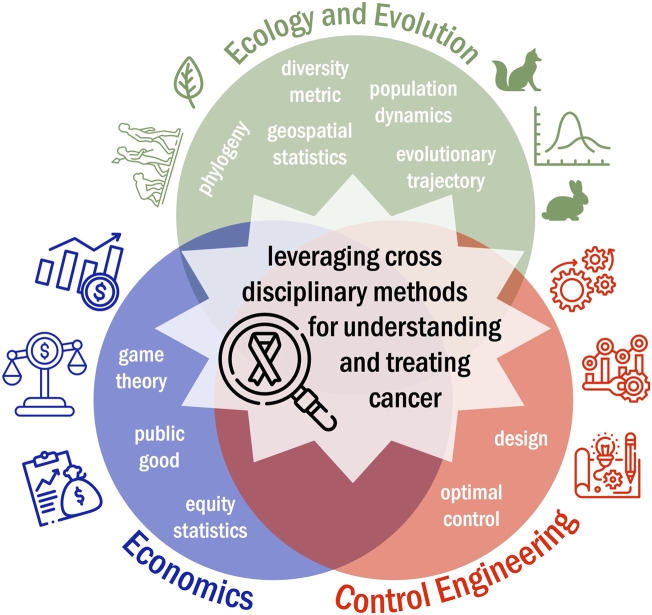
Pushing beyond computational systems biology, mathematical modeling frameworks from disciplines such as ecology and evolution, economics, and control engineering can be leveraged to better understand tumor progression and treatment response.

Here, we review the ways in which cancer researchers have leveraged cross-disciplinary mathematical modeling tools in ecology and evolution, economics, and control engineering to develop novel insights into the progression and treatment of adenocarcinomas (Figure 2). A search of the literature was carried out through PubMed and Embase databases. Key words used combinations of adenocarcinoma, mathematical, computational, *in silico*, ecology, evolution, evolutionary biology, economics, control theory. The search strategy followed a multi-stage approach implemented with the Covidence review management system where 628 unique manuscripts were identified by key word searches. Then, both authors screened all abstracts and shortlisted 176 abstracts whose text described principles from ecology and evolution, economics, or control engineering to answer cancer-related questions. In phase three, the full text was reviewed for all 176 manuscripts and 96 manuscripts were identified for inclusion in this review. These manuscripts were then subdivided and summarized based on field they drew insights from.

## 3 Ecology and evolution

Tumors are formed by multiple cell populations that interact and maintain an environment necessary for survival. Whenever conditions are altered, cancer cells can mutate or change phenotypes that allow them to adapt to this changing environment. These changes can contribute to the development of treatment resistance, ultimately hindering clinicians’ ability to treat cancer and resulting in poor patient prognosis. Therefore, understanding the evolutionary changes as a result of different environments and selective pressures that tumors experience can aid in the selection of effective treatment strategies (Beckman et al., 2020).

Like the canonical example from ecology of fox and rabbit population dynamics, where the fox behaves as the rabbit’s predator, cancer populations also face threats or predation due to immune cell populations or therapies. As the population size increases, these threats become more apparent, meaning that predation can be density dependent. Like with animal populations, some species work together for mutual benefit, cancer cells can also exhibit cooperative behaviors that promote tumor growth. Thus, cancer can be thought of as an ecological problem in multiple aspects: from tumor growth, metastasis and competition, which can be interpreted as the study of population dynamics in ecology, to spatial heterogeneity in the tumor microenvironment, which can be seen as the study of demographically structured populations in ecology (Reynolds et al., 2020). In this section, we will explore different mathematical modeling approaches from ecology and evolution that have been applied to study cancer progression and treatment response.

### 3.1 Population based modeling

A variety of tumor cell populations, such as treatment sensitive and resistant cells or supporting stroma and immune cells, have been modeled mathematically. In this section, we explored how researchers have studied the interactions between different cell populations using ecological modeling approaches such as Lotka-Volterra competition models. The most common approach to model the temporal dynamics of tumor populations is using ordinary differential equations (ODEs) or implemented as an agent-based model (ABM). ODEs describe changes in variables over a continuous time. The input are often population sizes, concentrations, or other continuous variables. On the other hand, ABMs simulate the discrete dynamics of a population of agents with specific rules regarding the behavior of interactions of the agents with other agents and their environment. Based on the discrete nature, ABMs often capture stochastic interactions in heterogenous systems and have often been applied to studying dynamics where spatial patterning is of interest.

One notable application of deterministic population dynamics modeling that applies ecological constructs to cancer research is the ODE model developed by Grassberger et al. that predicted the growth of drug-sensitive cells, drug-tolerant or drug-persistent cells, and drug-resistant cells to tyrosine kinase inhibitors (TKI) in EGFR-mutant lung cancer. The ODEs for the TKI-persister and TKI-resistant populations included a term that described the transition between the two phenotypes as well as an interaction term that explained the interplay between TKI-persistent and TKI-resistant cells. By using continuous lung tumor volume data abstracted from CT scans, they demonstrated that the growth rate of TKI-resistant cells was correlated to tumor progression (Grassberger et al., 2019). Cerasuolo et al. studied the evolution of TKI resistance using a more complex system of five differential equations describing the growth of sensitive and resistant cells, the changes in pharmacokinetics of a prostate cancer drug called enzalutamide, and the dynamics of the tumor microenvironment. In this model, the authors parameterized the ODE model using *in vitro* and *in vivo* data. Cell counts were collected *in vitro* from murine prostates adenocarcinoma cell lines cultures using flow cytometry. Tumor volume data was extracted from measuring *in vivo* mouse tumors. For both sets of data, their growth was assumed to follow logistic growth. Cerasuolo et al. also introduced a diffusion term to account for the random variation in cell number in the tumor microenvironment, also known as Brownian motion (Cerasuolo et al., 2020). The addition of stochasticity provided a more accurate representation of the behavior of the microenvironment in prostate cancer allowing the researchers to explore a second drug used in combination with enzalutamide. ODEs have also been used to model androgen-dependent and androgen-independent prostate cancer cells. Draghi et al. developed a system of equations that described the interaction between the health status of the patient including their ability to sustain treatment and develop resistance, the prostate-specific antigen (PSA) level produced by the androgen-dependent cells and androgen-independent cells, and the androgen level (Draghi et al., 2019). PSA levels collected from patients under intermittent therapy over time were used to parameterize this ODE model. The researchers were able to show that mathematically modeling PSA levels over time has the potential to predict treatment response and optimize treatment schedules.

When it comes to studying the interactions of different cancer populations that co-exist in the same microenvironment and compete for survival, Lotka-Volterra competition models are frequently applied. These models originate from ecology through studying predation in different animal populations, where one species acts as the prey whose population size is dependent on the rate of predation by the predator. These concepts have also been applied to model different cancers using ODEs that represent populations of cells interacting and competing for the same resources. The equations are usually modeled as logistic growth where total population growth is ultimately dampened by the availability of resources and some carrying capacity of the environment. However, different variations of the Lotka-Volterra models have been explored in order to fit the growth patterns of cell populations (Michelson, 1987; Michelson et al., 1987). Evolutionary-based therapy scheduling, also referred to as adaptive therapy, uses Lotka-Volterra interactions between cancer cell populations of interest to inform chemotherapeutic schedules. These schedules aim to reduce tumor sizes without allowing dominance of a treatment-resistant cell type by considering the dynamics between different cell types within the tumor. Adaptive therapy aims to move away from administrating maximum tolerated doses, which often result in the development of treatment resistance; to administering strategically timed chemotherapy doses. This aims to better control tumor size at lower cumulative treatment doses and allows for treatment ‘holidays’ for the patients, which may also reduce side effects patients experience (Belkhir et al., 2021). Lower cumulative drug doses determined by mathematical models allow for enough sensitive cells to still outcompete resistant cells for dominance in the tumor population. By delaying the development of resistance, sensitive clones can still be treated resulting on longer life expectancy. Lotka-Volterra based models are usually formatted as differential equations of pairs of treatment-resistant and treatment-sensitive populations or quiescent and proliferating populations and a term that captures the effects of the drug on the sensitive or proliferating population (Strobl et al., 2023). Adaptive therapy has shown to delay the onset of resistance of abiraterone in a clinical trial of metastatic castration-resistant prostate cancer (NCT02415621) where treatment decisions were made based on a mathematical model of androgen-dependent cells, androgen-independent cells, and testosterone producing cells with patient PSA level serving as a biomarker for tumor burden (Cunningham et al., 2020). This trial resulted in an increased of time to progression of 19.2 months with a 46% decrease in total drug dose compared to standard of care therapies (Zhang et al., 2017). Ongoing adaptive therapy clinical trials leveraging similar approaches have continue in prostate cancer and been applied to ovarian cancer patients (NCT05080556) (Draghi et al., 2019; Michelson, 1987; Gallaher et al., 2023). Outside of adenocarcinomas, adaptive therapy clinical trials have been developed for rhabdomyosarcoma (NCT04388839) (Reed et al., 2020), BRAF-mutant melanoma (NCT03543969), and basal cell carcinoma (NCT05651828) indicating that this treatment strategy could be applied to variety of cancer types.

Several ODE models have been used to study molecular changes within cancer cells exposed to different treatments as well as their effect on the evolution of resistance and overall changes in tumor size. These changes will cause the cells to adapt to new environments that lack certain biomolecules or compete with other populations for resources. This has been explored in both cervical and non-small cell lung cancer, where tumor volume has been analyzed in relation to the radiation fraction administered. Radiation can be modeled based on the number of oxygenated cells and hypoxic areas in the tumor, which are included in the ODEs as oxygenation rate constants (Chen et al., 2019; Belfatto et al., 2016). ODE models have also studied cancer progression regarding metabolic processes, in the absence of treatment. Voutouri et al. studied how osmotic pressures and mechanical changes in the microenvironment can affect tumor progression. They developed a model that incorporates the fluid and solid phase of the tumor, but also the transport of cation and anions on glycosaminoglycan chains. In this case, a deformation gradient tensor is used to quantify the three-dimensional changes in the tumor, which provides information about the degree of tumor deformation due to different elements (Voutouri and Stylianopoulos, 2014). A different research group developed a nutrient-depletion model, where they were able to study the interaction of leukemia and cervical cancer cells using population ecology concepts. Co-cultures experiments were developed to investigate whether proliferating cervical cancer epithelial cells can inhibit leukemia T-cells. The populations were modeled following prey-predation like interactions assuming that each cell type lived in a niche forming two different cell ecosystems that interact by exchanging molecules (Sega and Chignola, 2014). Studies in pancreatic and breast cancer have focused on tumor progression as it pertains to different metabolic conditions in the tumor microenvironment. These groups have developed systems of differential equations that explain changes on the amount of glucose, oxygen, pH, and other metabolite concentrations used in processes such as glycolysis, tricarboxylic acid cycle, or the pentose pathway (Roy and Finley, 2017; Damaghi et al., 2021). These studies allowed researchers to create *in silico* simulations of enzyme or element deprivation and evaluate the effects on cell proliferation. Specifically, oxygen levels in the tumor have been widely studied since the lack of this element in the tumor microenvironment leads to cancer invasion and metastasis. Oxygen levels, however, are most commonly modeled using linear models of diffusion and not differential equations (Milotti et al., 2020). Information obtained from using both approaches suggest different metabolic targets to slow the progression of certain cancers. Once again, mathematical modeling was able to predict cellular growth based on different metabolic processes.

While traditional ODEs have often been leveraged in population dynamics modeling of tumor ecology, some researchers hoping to capture the range of behaviors a tumor might exhibit under different selective pressures have turned to ABMs. This framework consists of individual agents, which are unique and autonomous cells or molecules that can interact with each other and their microenvironment following a set of specified rules and these frameworks often incorporate stochasticity and can include spatial resolved information. For instance, Deisboeck et al. developed an *in silico* ABM to simulate the possible effects of engineered cells in the progression of the tumor. In their model, the engineered cells were primary tumor cells from a patient modified to express higher proliferation rates than native tumor cells. They also carry an on-off switch that can be targeted therapeutically to induce apoptosis. The researchers hypothesized that the engineered cells could outgrow the native tumor cells and the final population could be eliminated using a therapeutic. Here, each cell (engineered or native) is an agent that can obtain ‘proliferation’ credits, which determine whether the cell will proceed to proliferation or remain in place allowing the other population to dominate in the environment. The simulations capture a 3D tumor microenvironment composed of an initial native tumor cell and an initial engineered cell, which has a higher proliferation rate compared to native tumor cells (Deisboeck and Wang, 2008). The simulations showed the efficacy of engineered tumor cells at controlling and potentially combating cancer once they reach a certain proliferation level. However, these engineered cells would have to be developed from primary tumor cells and subsequently implanted in close proximities to the tumor resulting in a highly invasive procedure for the patient. Maley et al. developed an ABM for the pre-cancerous condition Barrett’s esophagus, which can develop into esophageal adenocarcinoma. The researchers described cancer progression in three stages (normal, pre-cancerous, and malignant) based on the accumulation of neutral and selective mutations. The transition of these agents or cells between cancer stages was determined by the number of accumulated mutations and their ability to increase the mutation rates, as well as the time required for cell division. This research was able to provide insights on the number of different mutation types necessary for the development of malignancy (Maley and Forrest, 2000).

Population based modeling has been widely applied to adenocarcinomas given that it forms solid tumors, and temporal data such as tumor size, or population concentrations, can be obtained from CT scans, flow cytometry counts, and *in vitro* cultures. In this section, we have explored the usage two dynamics models: continuous models (e.g., ODE models) and discrete models incorporating stochasticity (e.g., ABMs) to obtain information about cancer progression. Deterministic models can provide continuous estimates of cancer behavior over time based distinct timepoint data. These models often use ODEs to describe systems that have well-defined parameters and known interactions. ODE modeling is a good platform to test out different mechanisms of action. In contrast, stochastic models include randomness or probabilistic elements into the system and thus need to be simulated many times to get the range of behaviors of the system of interest. Thus, these models are best suited to answer questions about behaviors such as the unpredictable nature of mutations, cell interactions, or treatment responses at low population sizes. Deterministic models are commonly used to predict average or bulk trends experienced by the populations of interest making their parameterization a computationally efficient process. Stochastic modeling requires additional input data and since it simulates random fluctuations it can be more computationally expensive. However, the use of stochasticity is not always appropriate when modeling cancer progression. Whenever populations are small, random mutations can greatly affect the development of that population and accuracy of the model. Mutations in large populations are not as impactful and could be left out of the model. Both deterministic and stochastic modeling approaches contribute to the understanding of the complex interactions in cancer progression, so their usage depends on the biological question, and available computational resources and data. Some researchers have also leveraged a hybrid approach that changes based on population size as demonstrated in modeling of immunotherapy treatment response in lymphoma (Kimmel et al., 2021).

### 3.2 Spatial ecology modeling

While the interactions between cell populations is important for cancer development, their spatial distribution can also play a critical role. Similar to animal populations distributed within an ecosystem, cellular populations in the tumor and its microenvironment are often arranged in specific patterns, which impacts the progression of the disease. What differentiates cancer from the populations commonly studied in ecology is the greater degree of spatial heterogeneity due to the presence of different cell types, phenotypes, and mutations. Hence, it is crucial to study the spatial distribution and clonal evolution patterns of these populations. Spatial data can be collected using different modalities including imaging, transcriptomic, or genomic techniques. Often, this data is collected from different areas in the tumor or from the primary tumor and distant mets and the expression profiles are compared. For instance, single-cell RNA sequencing has often been used to offer spatial context of cell locations in addition to transcriptomic data. Histological tumor samples have also been used to extrapolate cellular locations and predict treatment effectiveness based on cell distributions. Spatial ecology modeling ensures that therapies can effectively target the entire tumor and its microenvironment, rather than just a subset of populations (González-García et al., 2002; Yuan, 2016).

Spatial modeling approaches used in cancer not only deal with cell-to-cell interactions, but also with changes in cell shapes and sizes. Sapi et al. created a mathematical model to understand the changes in colorectal tumor volume under bevacizumab therapy. Their 2D model used a digital caliper to collect different measurements of the subcutaneous tumor under different bevacizumab concentrations without the need of performing an MRI. Measurements including tumor length and width were recorded for each mouse at least six times during the duration of the experiment. The authors showed that daily low doses of bevacizumab were able to cause a greater tumor volume reduction than a singular high dose (Sápi et al., 2015). Yamamoto et al. have also investigated effective treatment schedules and drug concentrations for pancreatic cancer, particularly with a combination of FOLFIRINOX and nab-paclitaxel therapy. They used time series tumor volume data from primary and metastatic site MRIs in a three cell-type logistic branching growth model. The growth was modeled starting with an initial cell that gives rise to proliferating cells that can accumulate mutations and transform to metastatic cells and establish metastatic sites. Yamamoto et al. introduced a quantifiable metric called Local Advancement Index (LAI), as an indicator of metastasis where the growth rate of the cells decreases as the tumor size increases. They concluded that tumors with lower LAI have a higher potential to metastasize and develop more metastases, (Yamamoto et al., 2019).

Volume changes have also been applied to ground-glass opacities, which are areas in the lung tissue that appear opaque. These opacities are indicative of various conditions including non-small cell lung cancer, especially when large volume alterations are observed. To understand the spatial-temporal dynamics of these opacities, Farkas et al. developed a model using diffusive logistic partial differential equations (PDE), which incorporated random movement of particles (Brownian motion). In this case, CT histograms, a graphical representation of the pixel intensities in CT region, were used as inputs for the model, which outputs a spatial density at a specific time. This information was used to identify doubling times of the ground glass opacities volume, serving as a marker of lung cancer progression (Farkas et al., 2018).

While the spatial location and volume of the tumor are important when selecting treatment strategies, the cell-to-cell interactions within the tumor can also play a role in determining cancer progression. Research groups such as Yang et al. have used both bulk and single-cell transcriptomic data to explore the presence and impact of tumor-associated macrophages on pancreatic adenocarcinoma development. They used CellPhoneDB (Efremova et al., 2020), a database analyzing cell-cell communications, to construct a network describing the interactions between macrophages and cancer cells. Additionally, the authors identified the presence of inflammatory macrophages in the tumor microenvironment as well as specific ligand-receptor pairs between tumor and immune cells (Yang et al., 2023). Information obtained from these networks provided a better understanding of macrophage interactions with pancreatic cancer providing an opportunity to develop targeted immunotherapies. Shan et al. also utilized spatially resolved bulk and single-cell transcriptomics data to detect the presence of stroma, cancer, normal pancreatic, and duct epithelium cells and their interactions in pancreatic cancer. The authors used SpaMOD (spatial molecular patterns) (Andres et al., 2001), a spatial modeling tool based on a partial least squares regression approach, to integrate spatially resolved transcriptomic data derived from the same pancreatic cancer tissue. By combining the two types of data, researchers identified the location of certain cell types within the tissue and their function. Understanding the spatial and functional organization of tissue has the potential to provide information about cancer stages and progression (Shan et al., 2022). Casesent et al. introduced a novel approach to collect spatially resolved genomic data called topographic single-cell sequencing, to study the invasiveness of ductal carcinoma in the breast. This innovative technique integrates laser-capture microdissection, laser catapulting, whole-genome amplification, and single-cell DNA sequencing to provide spatially resolved genomic data. After studying *in situ* and invasive ductal carcinoma samples, topographic single-cell sequencing identified a genomic transition from *in situ* to invasive phenotypes due to mutations. Thus the presence of mutant clones in the duct could indicate the potential development of metastasis (Casasent et al., 2018).

Understanding clonal evolution within a spatial context offers valuable insights into the selection and metastasis of distinct clones. The strength of selection can be measured using next-generation sequencing data (NGS), quantified as variant allele frequencies (VAFs). These allele frequencies represent the proportion of alleles at a particular locus that differ from the reference genome sequence. In cancer, the distribution of VAFs represent the genetic variants within the population. These distributions can be used to simulate spatial tumor growth models aimed at identifying mechanisms driving cancer progression, including the influence of natural selection and genetic drift. This methodology has been applied to multiple types of adenocarcinomas such as colorectal, lung, and esophageal (Sun et al., 2017). Utilizing variant information obtained from NGS, Sievers et al. identified that cancerous mutations can arise in small colon polyps. Modeling the molecular pathways involved in the growth of colorectal polyps and considering their mutation profiles, could offer insights in the clonal evolution of polyps that may progress into cancer (Sievers et al., 2017). In lung adenocarcinomas, studies aimed to uncover the clonal evolution of histomorphological patterns used global DNA methylation data, whole exome sequencing (WES), and RNA sequencing (Dietz et al., 2019; Karasaki et al., 2023). Dietz et al. focused on studying DNA methylation sites and the change in gene copy number in a phylogenetic framework to identify the origin of metastasis in the lymph nodes. Karasaki et al. developed a phylogenetic approach using information from changes in genomic distances that exists within different regional growth patterns in the tumor. By combining morphological, genomic, and clinical data, both groups concluded that even though different growth patterns were present within the same tumor, the most aggressive type or high-grade tumor patterns were associated with a high metastatic potential.

Spatial tumor information allows clinicians to identify the location of malignant clones and researchers often study how the genetic content compares between primary tumor and metastatic sites. Transcriptomic data (e.g., single-cell RNA sequencing data), epigenetic data (e.g., DNA methylation sites), imaging data (e.g., MRI), and genomic data (e.g., WES) have been used to retrieve the location and function of different cell types within a tumor. This data is often collected from different sections in the tumor or metastatic sites that can be compared at the transcriptomic, epigenetic, or genomic level. Again, the biological question becomes important when choosing a data type. Transcriptomic data provides a snapshot of the RNA information expressed by the cells, while genomics data can provide long-term view of gene regulation. However, when identifying and comparing mutations present in different areas of the tumor, genomic data would be necessary. Collecting all these data types is clinically expensive and the results can be complex to interpret. Nevertheless, clinicians could leverage spatial models to identify aggressive sites with metastatic potential and aim to target those initially.

### 3.3 Stochastic and probabilistic modeling

The deterministic modeling approaches presented thus far, neglect the stochasticity of random events including the well-known mutation accumulation that happens in many cancers. Therefore, some groups have focused on incorporating these stochastic elements when modeling cancer systems. While there are some known mechanistic progression pathways for certain cancer types, adenocarcinomas such as esophageal or prostate cancer can arise based on a random sequence of events. Aimed at improving screening techniques, researchers have modeled esophageal cancer as a Markov process. This random process delineates the order of different events in tumor progression, where the probability of each event happening only depends on the previous event. This model is suitable for studying esophageal cancer progression because it mirrors the typical patient experience: starting with gastroesophageal reflux disease, advancing to Barrett’s esophagus, followed by undetected cancer, detected cancer, and ultimately leading to death. The multistage Markov model that researchers have developed for esophageal cancer has been effective at determining the appropriate time for screening and starting treatment (Hur et al., 2010; Curtius et al., 2020; Curtius et al., 2021). Prostate cancer has also been modeled as a Markov process across different stages during disease progression. Peirolo et al. studied the transition between well, moderately, and poorly differentiated prostate cancer cells. These cell states were related to a lower and greater stage prostate cancer, respectively. This model estimated cancer progression and identified the grade of the disease using clinical data including survival probability, frequency of detection given a grade and a volume, and probability of metastasis (Peirolo and Scalerandi, 2004). Gastric cancer can also transition through increasingly malignant states from normal gastric mucosa, to chronic nonatrophic gastritis, gastric atrophy, intestinal neoplasia, dysplasia, and finally to gastric cancer. Most of these transitions are determined by a bacterium called *H. pylori*. Yeh et al. developed a Markov transition model, where an empirical calibration approach was used to estimate good-fitting parameters in line with epidemiologic data obtained from the literature. This model aimed to explore the relationship between *H. pylori* and gastric cancer to develop strategies for cancer prevention (Yeh et al., 2008).

While many studies have focused on the different stages of cancer as their basis for their Markov model, Veestraeten et al. developed a continuous-time stochastic process for prostate cancer progression based on the variations of the prostate-specific antigen (PSA). This stochastic variable reached a lower boundary after therapy, where progression of the disease stopped. However, during relapse, the PSA level increased. The differential equation model created by the researchers considers a Wiener process, also known as Brownian motion, able to describe the stochastic nature of cancer progression. The parameters included in the model explained how PSA levels were affected by the growth of the tumor cells, and cell death due to various treatment intensities. This approach can help create personalized treatment schedules and track the effects of therapy in the progression of the disease (Veestraeten, 2006).

Another open question in cancer research focuses on the stochastic differentiation trajectories that cells may have travelled along to develop into cancer. Lopez et al. investigated the effect of harmful mutations on cancer evolution by examining whole genome doubling. The authors analyzed sequencing data from lung and triple-negative breast cancers to identify the timing of mutations relative to whole genome doubling. The model used a Gillespie algorithm, a stochastic method leveraged here to created simulations of cancer progression based on mutation rates, the sizes of driver and passenger mutations as well as their effect on fitness (López et al., 2020). Lopez et al. aimed to investigate stochastic mutational events in cancer genes that could be suppressed. Similarly, Lakatos et al. developed a stochastic branching process of tumor evolution to evaluate the production of neoantigens, specific proteins produced by the tumor. A kinetic Monte Carlo algorithm was used to simulate the evolution of colon, stomach, and endometrial cancers and their associated antigen production. In this case, identifying these mutated proteins could be targets for immunotherapies. However, there are still challenges when it comes to identifying the appropriate therapy based only on tumor size and neoantigen presence (Lakatos et al., 2020).

The presence of certain proteins or biomarkers in a tumor microenvironment can inform effective treatment strategies. Several groups have focused on developing molecular functional networks to identify possible interactions between different molecules that exist in the microenvironment. For instance, Chen et al. investigated the presence of biological molecules in lung adenocarcinomas by analyzing a network of genes, microRNA (miRNA), and long non-coding RNA (lncRNA) data linked to various stages of lung cancer. They utilized random walk and Monte Carlo algorithms to group these biological molecules, selecting certain clusters as modules due to their significant association with biological functions. These selected modules, along with their primary biological functions, were integrated to form a core evolution network. This network depicted the progression of lung cancer based on the biological molecules and their functions across the four disease stages (Chen et al., 2022). Jiang et al. also studied lung adenocarcinomas and the point of transition from epithelial to mesenchymal phenotypes in those tumors. Using gene expression data from lung adenocarcinoma cells where the protein TGF-beta has been known to induce epithelial to mesenchymal transition (EMT), researchers developed a Dynamic Network Biomarker (DNB) model. This model identified the timing and components of the EMT transition by examining high variation in the expression of DNB genes (measured as a coefficient of variation), strong correlation between DNB members (measured as the Pearson correlation coefficient), and weak correlation between DNB members and non-members (also measured as the Pearson correlation coefficient). These metrics were combined to calculate a composite index indicating the stage of DNB that led to the transition tipping point. SMAD7 and SERPINE1 were found to be promoters of EMT in lung adenocarcinomas (Jiang et al., 2020). While these dynamic networks can be used to provide insights into cancer staging, they can also inform patient survival. Fang et al. developed a survival network called ‘dnet,’ which uses gene mutations and survival data from breast, colon, lung, and ovarian adenocarcinomas to identify which gene mutations were related to patient survival. Briefly, dnet used TCGA mutation and survival data from cancer patients. This data included mutation frequencies across patients for different genes, along with three clinical variables: age, gender and tumor type. Dnet conducts survival analysis based on the Cox proportional hazards model to evaluate the influence of mutations on patient survival. The Cox proportional hazard ratio (HR) and the associated *p*-values for each gene were calculated using a likelihood ratio test between the full Cox regression and the baseline regression, identifying potential predictors for various cancer types. Lastly, a patient-survival gene network was constructed from the highest-scoring subgraph determined by the Cox proportional HR *p*-values previously calculated. This network was used to identify different genes related to patient survival, which could be potential therapeutic targets in almost any type of cancer (Fang and Gough, 2014).

### 3.4 Phylogenetic analysis

Phylogenetic analysis and the development of phylogenetic trees have been widely applied to understand cancer progression and evolution. Starting from genomic or transcriptomic data, phylogenetic trees can be reconstructed using various criteria, revealing different aspects of tumor evolution. Many of the papers reviewed used next-generation sequencing (e.g., WES, whole-genome sequencing (WGS), DNA sequencing, RNA sequencing, cytogenetic data) data to unravel copy number variations (CNV) or single nucleotide variants (SNVs) driving disease development, which inform the emergence of new branches in phylogenetic tree models (Turajlic et al., 2015). Both CNVs and SNVs are common types of genetic alterations that appear in cancer. CNVs are changes in the number of copies of a specific segment of DNA, which leads to the amplification or deletion of genetic material. On the other hand, SNVs represent changes in single nucleotides in the DNA such as substitutions, insertions, or deletions. Both variations affect gene expression, and protein function, leading to the development and progression of cancer. The detection of these genetic alterations in patients contributes to the construction of phylogenetic trees that can inform cancer pathogenesis and the identify potential therapeutic targets. To study these alterations and reconstruct tumor phylogenesis, researchers have used the following algorithms: Bayesian or stochastic approaches, tumor clonality, maximum parsimony algorithms, clustering methods, or a hybrid method. While established tree construction algorithms such as BEAST or CLONET have originated from using these approaches, other algorithms are still in development. The process of creating new phylogenetic algorithms is closely tied to availability of data for the cancer and treatment of interest. Here, we aim to explore how traditional and new phylogenetic methods have been used to study cancer evolution and identify gene targets that might slow cancer progression and prevent emergence of resistance.

#### 3.4.1 Bayesian or stochastic approaches

Bayesian and stochastic approaches are powerful tools for constructing phylogenetic trees that incorporate probabilistic models and consider uncertainty. Due to the random events in cancer development and progression, these methods provide a more realistic representation of tumor evolution. These approaches also account for complex evolutionary processes including tumor heterogeneity and clonal dynamics. Bayesian phylogenetics uses Markov chain Monte Carlo (MCMC) models to reconstruct evolutionary processes by considering probability distributions. These models combine prior likelihood and prior probability of a tree to create posterior probability trees (Nascimento et al., 2017). Kostadinov et al. used a traditional algorithm called BEAST (Bayesian Evolutionary Analysis Sampling Trees) on somatic genomic abnormalities (SGA), including CNVs and SNVs that occur across somatic cells, from esophageal carcinoma. They investigated whether non-steroidal inflammatory drugs would reduce the rate of SGA and improve patient outcomes. BEAST was run for ten million iterations of the Bayesian MCMC algorithm to optimize model parameters fit to whole-genome SGA data and generate posterior phylogenetic distributions. The researchers modified the algorithm to connect the last universal common ancestor, which did not have any altered genomic states, with the most recent common ancestor. BEAST generated trees revealed that non-steroidal inflammatory drugs can reduce the frequency of SGAs in specific branches of the phylogeny (Kostadinov et al., 2013).

BEAST was used to infer the time of divergence of evolutionary events using prior tree distribution and parameters obtained with MCMC model. Similarly, another Bayesian MCMC algorithm called BAMSE (Bayesian Model Selection for Tumor Evolution) used prior information to perform tree reconstruction. While the methodologies of BAMSE and BEAST are similar, they differ on the likelihood functions used to incorporate prior information, and BAMSE offers flexibility to accommodate various types of datasets. BAMSE takes somatic mutation read counts as inputs and clusters them into scored subclones. For these subclones, different potential trees able to explain their evolutive patterns were created. The most plausible tree was then selected based on a Bayesian model that considers prior information from the subclones to calculate a posterior probability. This approach was particularly useful when reconstructing phylogenies for heterogeneous tumor samples or multiple samples from the same patient (Toosi et al., 2019).

Another Bayesian model called Treeomics has been developed to study the phylogenetic evolution of metastatic subclones and their seeding locations (Reiter et al., 2017). Treeomics has been used to investigate cancer metastasis in pancreatic, ovarian, and prostate cancers as well as precursor lesions. In pancreatic cancer, for instance, pancreatic intraepithelial neoplasia is a well-known precursor lesion, whose evolutionary pattern was able to be reconstructed with Treeomics (Makohon-Moore et al., 2018). Narrowing the focus, a research group developed SCITE another Bayesian probability algorithm using the beta-binomial distribution that can only be applied to single-cell DNA sequencing data. Leung et al. use this approach to reveal clonal lineages of liver metastases in colorectal cancer (Leung et al., 2017). All of these Bayesian inference models (BEAST, BAMSE, Treeomics, and SCITE) are able to construct tree phylogenies for genetic and transcriptomic data. SCITE is the only algorithm that retrieved phylogenies using information at a single-cell resolution while the other algorithms were applied to different types of genomic data. The main challenge with using phylogenetic reconstruction to inform cancer evolution and treatment is data availability. It is challenging to obtain allele frequencies from clinical data, such as formalin-fixed or paraffin-embedded tissue samples, so new sequencing techniques are being developed to leverage these approaches in the clinic. Additionally, most of these algorithms require user input of certain parameters that are difficult to estimate which could lead to erroneous phylogeny formations.

#### 3.4.2 Maximum parsimony algorithms

Most phylogenetic trees are generated based on maximum parsimony algorithms, which aim to create the least complex tree to explain the given data. Maximum parsimony phylogenetics is a non-probabilistic method that aims to uncover the molecular heterogeneity of diseases by minimizing the number of evolutionary changes. These algorithms have been applied to different types of data such as WES, CNVs, or cytogenetic data, however they are adjusted based on the data inputs. Li et al. used the maximum parsimony algorithm on WES data to understand the mutational landscape of subsolid nodules in lung adenocarcinoma (Li et al., 2020). Similarly, Nalbantoglu et al. analyzed WES data to identify pancreatic ductal adenocarcinoma genetic signatures and pathways that were associated with survival. While parsimony phylogenetics aim to unravel driver and passenger mutations associated with the disease, they could not identify a specific clone related with cancer survival (Nalbantoglu et al., 2016). An improved version of the parsimony approach was developed by Murugaesu et al. called the parsimony ratchet, which introduces randomness to the initial maximum parsimony tree created to improve the final tree formation. The researchers found how changes in the genomic landscape affected the evolution of esophageal carcinoma before and after neoadjuvant chemotherapy (Nixon, 1999; Murugaesu et al., 2015).

Maximum parsimony algorithms will always try to find the tree topology that requires the fewest evolutionary changes to explain the input data. A more generalized approach called maximum likelihood algorithms aim to find the tree topology that maximizes the probability of observing the given sequence under a specific evolutionary model independent of its simplicity. Heydebreck et al. used maximum likelihood estimation to create phylogenetic trees using cytogenetic data from clear cell renal cell carcinoma samples. In this model, genetic alterations were represented as branches, with a normal cell serving as the root of the tree. The tumor evolution was then traced starting at the root and assigning probabilities to genetic changes occurring at each node on the tree. The final tree revealed different genetic changes during tumor evolution (von Heydebreck et al., 2003). Petersson et al. studied chromosomal alterations and mutational variants related to poor prognosis in pancreatic cancer. They used a similar maximum likelihood algorithm where the root was a normal cell. In this case, CNVs, SNVs, and insertions and deletions were grouped based on their mutated clone fraction to identify mutations that only affected one subclone. This information was input into an event matrix, which the maximum likelihood algorithm model used to create the phylogenetic trees (Petersson et al., 2022).

When it comes to analyzing molecular data, GARLI (Genetic Algorithm for Rapid Likelihood Inference) is a maximum likelihood algorithm that was able to simulate genetic evolution and select the most likely tree topology through a process similar to natural selection. Zhao et al. utilize this algorithm on genomic DNA data from lung and pancreatic cancers to understand tumor progression of primary and metastatic lineages at the time of diagnosis (Zhao et al., 2016).

Dollo parsimony is a different type of parsimony that is characterized by the inability to regain traits once they have been lost in the phylogeny. McPherson et al. used this probabilistic model approach where SNVs could transition between two states, starting from an ancestral state to the next state but not switch back. This technique was applied to WGS and single-nucleus sequencing data in ovarian cancer, identifying different evolutionary features such as gain or loss mutations characteristic of disease evolution (V Alekseyenko et al., 2008; McPherson et al., 2016). Wu et al. also used Dollo parsimony, in a tool called Dolpenny, where somatic mutations and loss of heterozygosity data from serous tubal intraepithelial carcinoma (the precursor lesion of high-grade ovarian cancer) were converted to binary inputs to determine the most parsimony tree. They showed that the clonal origin of these lesion was diverse between patients and also within the same patient with multiple lesions (Wu et al., 2019).

Here, we have explored three different algorithms, maximum parsimony, maximum likelihood, and Dollo parsimony, used to reconstruct evolutionary topologies in cancer. Maximum likelihood algorithms use the given data and a specific model of evolution to construct a tree topology probability. The final tree would be the one that maximizes this probability while maximum parsimony algorithms minimize the number of evolutionary events on the final tree. Dollo parsimony introduces an additional simplification where traits cannot be regained once they are lost. As expected, maximum likelihood algorithms can be more accurate at retrieving phylogenetic progression, but they are more computationally expensive. Additionally, if the tree topology that best explains the data is very complicated, maximum likelihood algorithms might not be able to retrieve it. Contrarily, maximum and Dollo parsimony algorithms will be able to retrieve a phylogenetic tree in a shorter time, but they might lack of essential evolutionary information in the tree.

#### 3.4.3 Tumor clonality

The evolution of different clones in the tumor, their frequencies, and hierarchical organization is crucial for understanding cancer progression under different treatment conditions. Multiple research groups have developed algorithms such as CITUP, CLONET, and SCHISM that aim to identify the clonal evolution trajectories in cancer using multiple samples from the same patient. CITUP (Clonality Inference in Tumors using Phylogeny) was developed to study subclones that give rise to intra-tumor heterogeneity. This algorithm uses deep sequencing data processed by Quadratic Inference Programming (QIP) to determine phylogenetic trees and clonal frequencies. QIP handled large number of mutations in a fast manner to provide information on cancer heterogeneity (Malikic et al., 2015). Abbosh et al. used this approach on multi-region WES data from circulation tumor DNA to understand the evolutionary dynamics of early-stage disease in lung cancer (Abbosh et al., 2017). Similar to CITUP, CLONET (CLONality Estimate in Tumors) also aims to study the clonal structure of tumors based on somatic aberrations obtained from WGS, which includes information from both coding and non-coding regions of the genome. This algorithm uses a local optimization approach to ensure tumor purity in noisy samples to estimate the high confidence values for driver genes that are related to the nodes of the tree (Prandi et al., 2014). Van der Mijn et al. used CLONET to understand the genomic landscape of clear cell renal cell carcinoma using WES and CNVs associated with worse prognosis (van der Mijn et al., 2022). SCHISM (Subclonal Hierarchy Inference from Somatic Mutations) employed somatic mutation counts obtained from NGS as inputs. Initially, these mutations were clustered and then a generalized ratio test was used to infer the temporal ordering of these mutations. This information was later used to construct phylogenetic trees (Niknafs et al., 2015). Researchers have used SCHISM to identity the type of evolution in pancreatic cancer mouse models. The evolutionary patterns identified in the mouse models were found to be very similar to human pancreatic cancer models (Niknafs et al., 2019). Clonality algorithms offer several advantages compared to maximum parsimony and Bayesian approaches. Algorithms such as CLONET, CITUP and SCHISM, can process large-scale data obtained from high-throughput sequencing technologies resulting in improved tree topology accuracy. They can also integrate biological knowledge and clinical data to provide additional information about the final tree such as clonal frequencies or accurate branching patterns. However, data availability to use these algorithms is scarce and it is expensive to collect.

#### 3.4.4 Clustering methods

Most the algorithms previously described consider genomic abnormalities including mutations, aberrations, or variants. However, regional or evolutionary distance data can also be used to create tree topologies through different clustering methods. Saitou et al., for instance, developed a neighbor-joining method where a neighbor was defined as a pair of operational taxonomic units (OTU) that were connected through one interior node. Their algorithm initially created a star-like tree with the different OTUs and then paired them into neighbours with the smallest sum of branch lengths (Saitou and Nei, 1987). This algorithm was applied to circulating tumor DNA data in colorectal cancer to understand the evolution of metastases under HER2 blockade treatment. In this case, primary tumor data formed the root of the tree and the branches showed the different spatial subclones that could potentially develop resistance to the treatment (Siravegna et al., 2018). Matsui et al. developed another algorithm called phyC to create evolutionary trees based on multi-regional sequencing. This algorithm clusters a set of trees based on the VAFs and the evolutionary trees obtained were divided by subgroups based on different topologies and edge characteristics. These trees informed the different evolutionary patterns that can exist during disease development (Matsui et al., 2017). To address sample heterogeneity in the reconstruction and clustering of lineages, Popic et al. developed LICHeE (Lineage Inference for Cancer Heterogeneity and Evolution). This algorithm identified somatic single nucleotide variants (SSNVs) groups from germline SNVs and VAFs obtained from different cancerous cells locations (primary tumor, metastases, lymph nodes). The groups were aggregated by their VAFs, forming clusters. These clusters were then used to create an evolutionary constraint network, starting from the germline cluster, and connecting each cluster based on their VAFs quantity, ensuring that the parent node had a higher VAF than the child node. The evolutionary trees were inferred from this constraint network. LICHeE has been applied in ovarian and breast cancer data, successfully replicating and improving tree topologies previously obtained by other researchers, revealing the heterogeneity among multiple somatic samples based on SNV and VAF data (Popic et al., 2015). Clustering algorithms can often be more computationally efficient and can capture complex evolutionary patterns more effectively due to their clustering-based approach. Like other phylogenetic approaches, data availability becomes a challenge, preventing the use of this approach to inform routine treatment decisions.

#### 3.4.5 Hybrid approaches

Researchers have also combined multiple of these approaches to reconstruct more complex phylogenies. El-Kebir et al. developed an algorithm called CNT-ILP (Copy-Number Tree-Integer Linear Program) to solve a specific problem of copy-number triplet and the copy-number tree when using copy-number profile data. The copy-number triplet problem arises when the algorithm is incapable of find a parental tree that minimizes the distances to its children due to the continuous range of copy number states in CNVs. The copy-number tree problem arises when the algorithm is incapable of creating the simplest tree under maximum parsimony. These two problems were addressed using a pseudo-polynomial time algorithm and an integer linear program (ILP). These methods treated the problems as optimization tasks, enabling the consideration of copy-number states in the reconstruction of phylogenies (El-Kebir et al., 2017). Fimereli et al. utilized this optimized algorithm to analyze primary and metastatic copy number profiles from breast cancer. This approach decreased the computational time, enabling the researchers to gain insights into the metastatic behaviour of the cancer. (Fimereli et al., 2022).

Phylogenetic trees can also be constructed based on cells’ ploidy, which can vary from diploid, typical of most human cells, to lead to aneuploidy or polyploid populations resulting from cancer mutations. Gertz et al. leveraged fluorescence *in situ* hybridization (FISH) to detect changes in the ploidy of tumors using a probe and mixed integer linear programming (MILP) to create FISHtrees 3.0. This algorithm was validated using data from breast ductal carcinoma *in situ* and invasive ductal carcinoma (Gertz et al., 2016). A different group improved phylogenetic tree reconstruction by creating CONIPHER (Correcting Noise In Phylogenetic Evolution and Reconstruction). This algorithm is able to handle a high number of primary tumor and metastatic regions per patient, corrects for different evolutionary events such as mutation losses and removes clusters that were unlikely to happen biologically. Initially, CONIPHER identified clusters based on the somatic mutations that occurred in different subclones during evolution using the algorithm PyClone (Roth et al., 2014). The next step involved the creation of a phylogenetic tree using the identified mutation clusters and eliminating false clusters originated from artefactual mutations or somatic copy number aberrations. The last step consisted of enumerating plausible phylogenetic trees. CONIPHER has studied the timing of somatic events in lung cancer tumors from WES (Frankell et al., 2023).

Phylogenetic algorithms have been useful in cancer research to explore complex evolutionary patterns during tumor evolution. While some approaches might be able to utilize more information than others, they highlight potential therapeutic biomarkers. Regardless of the mathematical concepts involved in each approach, the construction of phylogenetic topologies is limited by data availability. These algorithms require genomic data, including CNVs and SNVs, to able to estimate the appropriate branching structure of the phylogenetic tree. This data is not collected for every patient, but if it is, it only represents a snapshot of the tumor at the specific point in time. Thus, phylogenetic information is useful for researchers investigating biomarker targets at certain disease stages but may not be as helpful in a rapidly evolving tumor.

### 3.5 Diversity measures

Researchers have also leveraged different diversity indexes from ecology to quantify cellular and phenotypic heterogeneity in tumor samples. These can help assess the richness, evenness, and dominance of different cell types within a tumor sample. Richness refers to the amount or count of cell types present in a sample, while evenness represents the abundance of a certain type compared to the total. Dominance describes the type with the largest influence and control of the overall population. The Shannon index (Shannon, 1948) is the most commonly used metric to quantify diversity within a dataset, considering the uncertainty associated with the presence of specific cell types. A more general approach is leveraging the generalized diversity index (GDI) (Jost, 2006), which can identify diversity across a number of scales of diversity within a dataset (Roswell et al., 2021; Crupi et al., 2019). These indexes have been applied to various cancer types. For instance, in clear-cell renal cell carcinoma, GDI was used to evaluate immune cell infiltration from patient bulk RNA sequencing data. Additionally, in patients with chronic myelomonocytic leukemia (CMML), researchers use the Shannon index to quantify cytokine receptor diversity in different stem and myeloid progenitor populations from patient samples. Higher Shannon indices were associated with an increase in the cytokine receptor diversity in hematopoietic stem cells in patients with CMML compared to healthy patients (Ferrall-Fairbanks et al., 2022a). While the Shannon index has been informative, the GDI emerges as a more robust metric. GDI is constructed as a continuous and non-increasing function within a range of values that are determined by the scale parameter q, which is the order of diversity (Crupi et al., 2019). Lower order metrics favor clonal richness, whereas higher order metrics give greater importance to the most abundant population. Ferrall-Fairbanks et al. first applied GDI to quantify levels of heterogeneity in cancer from single-cell RNA-sequencing data, extracting insights into cancer evolution (Ferrall-Fairbanks et al., 2019). GDI was also used to study parasexual recombination in breast cancer, a process involving the exchange of genetic material between cells without meiosis, where 2 cell lines fused to produce hybrids. Miroshnychenko et al. showed that at early passages, hybrids have a higher GDI across all orders (values of q) compared to the parental cell lines. At later passages, the GDI decreased at low orders, meaning that the species richness decreased, while at high orders was still high, which suggests that the species evenness in the hybrid cells was higher than the parental cells (Miroshnychenko et al., 2021). GDI was also applied to quantify immune cell infiltration using CDR3 sequences recovered from bulk RNA-sequencing of clear cell renal cell carcinoma samples. The authors showed that larger and more advanced disease stage cancers have increased richness in tumor recovered CDR3 sequences, while the evenness of these distributions segregated patients based on survival (Ferrall-Fairbanks et al., 2022b). As seen in this section, the diversity indices can be useful to assess the heterogeneity in a patient population based on clinicopathologic features, which could predict disease progression and treatment response.

## 4 Economics

Studying the economic burdens in cancer patients has been of interests for researchers worldwide. However, here we have shifted the focus towards presenting mathematical principles originated from the field of economics that have been then applied to study cancer progression and treatment response. One of these concepts is game theory, or evolutionary game theory (EGT) when applied to biological problems, which describes interacting populations in terms of strategies and pay-offs. In this sense, EGT relates the dependence of cells to each other, where the fitness of 1 cell depends on their surrounding populations and/or environment. While cancer is typically studied in the context of natural selection, also known as the Darwinian process, incorporating elements of Darwinian evolution into game theory model can provide important new insights. Therefore, evolutionary elements such as mutations or chromosomal rearrangements, competition for resources, and the effects of different traits on survival are key elements when modeling these types of problems. In this evolutionary game, the cancer cells would be the players, the inherited traits are the strategies, and the payoffs usually correspond to the fitness and survival of the cells. However, each research group can model their evolutionary game differently depending on their specific questions (Wölfl et al., 2022; Laruelle et al., 2023; Stanková et al., 2019). EGT has been used to model cancer resistance, interactions between different cell types in the tumor microenvironment, or the emergence of different cell states. Researchers have used both spatial and non-spatial datasets to apply this framework, while maintaining the overall goal of unravelling cancer evolution based on cellular interactions (Coggan and Page, 2022). Furthermore, an economic concept known as the Gini index was developed to identify wealth inequality and can be used as a metric to quantify population inequalities in cancer populations.

### 4.1 Game theory

EGT has been used to study cancer progression by identifying different players and the payoffs in a cancer system, often the tumor microenvironment. Archetti et al. studied a group of engineered cell populations as a public goods game. In their framework, they engineered cancer cells by knocking-out genes that produce growth factors needed to live and proliferate. By combining non-engineered and engineered cancer cells *in-vitro*, the overall population growth decreased compared to non-engineered cancer cells alone. This occurred because the engineered cancer cells disrupted the cooperative nature of the population by consuming growth factors (public goods) produced at a cost by the non-engineered population. This cost led to complete or almost complete eradication of the main growth factor-producing clone, resulting in a reduction in population growth. This model was applied to different types of cancer cells such as non-small cell lung cancer and pancreatic neuroendocrine cancer (Archetti, 2021).

EGT has often been paired with some of the methodologies already described. Malekian et al., for instance, created a model that combines EGT concepts with an ABM to study the effects of gap junctions in ductal carcinoma *in situ* progression. The agent-based portion of the model considers each cell to exist in one of the following states: quiescent, proliferative, apoptotic, and necrotic. These cells can transition between states based on oxygen concentration and the phase of the cell cycle. The EGT portion of the model was designed to address the interactions between cells. Here each agent, or cell, represents a player that can interact with any of the 8 cells that surrounds it using one strategy at the time (quiescence, proliferation, apoptosis, and necrosis). In their model, the interaction between 2 cells through gap junctions created a pay-off that sends the cell a survival or death signal based on different factors in the microenvironment such as oxygen levels or growth factors concentrations (Malekian et al., 2016).

Zhang et al. combined EGT concepts with a Lotka-Volterra model. The authors aimed to explain the competition of different cells in metastatic castrate-resistant prostate cancer under adaptive therapy. Their players were the cancer cells, the strategies the different phenotypes that a cell can take, in this case androgen-dependent, testosterone-producing, or androgen-independent cells, and lastly the payoffs were proliferation and survival of the cells. The Lotka-Volterra model addressed the competition of the three different cell phenotypes while undergoing therapy (Zhang et al., 2017).

Spatial data can also be modeled using game theory. Kaznatcheer et al. defined cancer cells as players that can take one of two different strategies: those exhibiting autonomous growth that are capable of rapid proliferation (AG cells) and those with acquired motility and invasiveness conferred by mutations (INV cells). These phenotypes were tied to different payoffs: the cost of motility on INV cells, and the benefit of fitness of a cell that has every resource available. For example, if two INV cells meet, then one gets the benefit of using the surrounding resources and the other one migrates at a cost. However, if a INV cell encounters an AG cell, then the INV cell pays the cost of having to move to find resources, while the AG benefits from that interaction. Using data from prostate cancer, researchers were able to justify that the spatial location of the cells can affect tumor invasion and evolution based on these cellular interactions (Kaznatcheev et al., 2015).

### 4.2 Gini index

The Gini index was originally developed as a measure of economic inequality. In the context of cancer, it can be used to quantify cellular heterogeneity within the tumor to predict disease progression and treatment response. Hinohara et al. used the Gini index to quantify heterogenic expression of specific markers in breast cancer. Specifically, this index was used to identify variations in the expression levels of a group of enzymes (KDM5) implicated in cell proliferation and cancer progression in breast cancer cell lines, both pre- and post-treatment with an inhibitor targeting that protein family. They concluded that inhibiting KDM5 enzymes decreased transcriptomic heterogeneity, which could increase treatment effectiveness (Hinohara et al., 2019). Also in breast cancer, Filho et al. used the Gini index to quantify heterogeneity of ERBB2 gene expression in HER2 positive patients. In this type of cancer, an intratumor heterogeneity expression in the ERBB2 gene is associated with cancer resistance. However, the researchers observed a relatively low Gini index, suggesting that the samples have an evenly distributed expression level of the ERBB2 gene. In this case, the Gini index was not a good predictor of drug response (Filho et al., 2021). This index can also be used to quantify expression of markers that suggest response or resistance to treatment. Gil Del Alcazar et al. used the Gini index to quantify changes in immune populations between stable and growing tumors in a breast cancer rat model. Stable tumors expressed a higher Gini index with respect to their B-cell and T-cell receptors. This implied a higher diversity in immune response making these tumors not suitable for immune therapies (Gil Del Alcazar et al., 2022).

The Gini index is usually used in combination with other diversity metrics. Miroshnychenko et al. used the GDI to quantify diversity between parental breast and epithelial cell types, and hybrids of the two, which could have been formed through cell fusions also known as somatic hybridization. While the GDI can differentiate cells into different phenotypes, the Gini index can capture cell-to-cell phenotypic variability. Therefore, the Gini dispersion index provided information about the gene expression variability of cells within the same phenotype. The researchers found that hybrid cell populations had higher Gini indexes compared to the parental populations indicating that somatic hybridizations increased phenotypic diversity (Miroshnychenko et al., 2021).

The application of economic mathematical principles has been useful to study cancer in several ways. Game theory has been able to explain different phenomena happening during cancer development such as resistance, cell interaction, and the emergence of different cell states. These processes were modeled as a game with players and pay-offs tied to different strategies. Game theory, in combination with ecology tools, has been able to capture additional cellular interactions creating a more robust framework to explain temporal data. Additionally, the Gini index originally developed to measure economic inequality, has been applied to quantify cellular diversity in tumors. Exploring the cellular composition indicates effectiveness of treatment. For instance, high diversity in immune populations within a tumor indicate that immunotherapy treatment would be ineffective. Data types such as population counts, or volume are necessary to utilize game theory models while sequencing data would be necessary to obtain a Gini index. Once again data availability becomes a challenge when using these tools to study cancer progression or treatment effectiveness.

## 5 Control engineering

Control theory has numerous applications in the fields of control engineering and mathematics. The objective of this theory is to control different systems and machines to obtain the most optimal performance. However, this framework can be applied to study cancer evolution as a system that can be controlled until a certain criterion is met. These controls can be variables in an ODE system that causes the system to minimize a certain process such as the development of treatment resistance or overall tumor growth/size (Lecca, 2020). Cecile Carrere et al. developed a control system to study cancer resistance in an adaptive therapy context. In their experiments, they analyzed the growth of both sensitive and resistant populations in a co-culture of a lung cancer cell line using ODEs. To transform their population dynamics model into a control problem, they incorporated a control mechanism using a Lebesgue measurable function. This function introduced measures of length, area, and volume to the system, allowing the optimization process to regulate and minimize the final tumor size when exposed to different treatment schedules (Carrère, 2017). Jonsson et al. also studied cancer resistance using control theory, focused on the switch of different treatment strategies to prevent resistance development. In their model, an equation described the growth, mutations, and evolution of a population of non-small cell lung cancer cells. Additionally, they considered a cost function that addressed the effectiveness of a specific treatment over time. This cost function tried to address whether it would be more optimal to continue with the same treatment to keep the tumor population at a minimum or to switch the treatment strategy to a different drug, or schedule. Using non-small cell lung cancer cellular dynamics, the group was able to simulate population growth under different treatment strategies (Jonsson et al., 2017). Similarly, Plaugher et al. developed a control model for different cellular populations involved in pancreatic cancer. In this case, each cell type had a different control influencing its growth such as treatment drugs, cytokines, or growth factors. With this framework, researchers took into consideration how different controls affect cellular populations individually instead of one control affecting the entire population (Plaugher and Murrugarra, 2021).

Control engineering is still under development when applied to cancer. Researchers are exploring various control strategies, optimization techniques, and mathematical models that could be useful to improve therapeutic outcomes. Modeling cancer as a control problem could provide precise manipulation of treatment strategies such as dosage, timing, or combination therapies. Contrary to game theory which is more accurate at capturing cell-to-cell interactions.

## 6 Discussion

**FIGURE 3:**
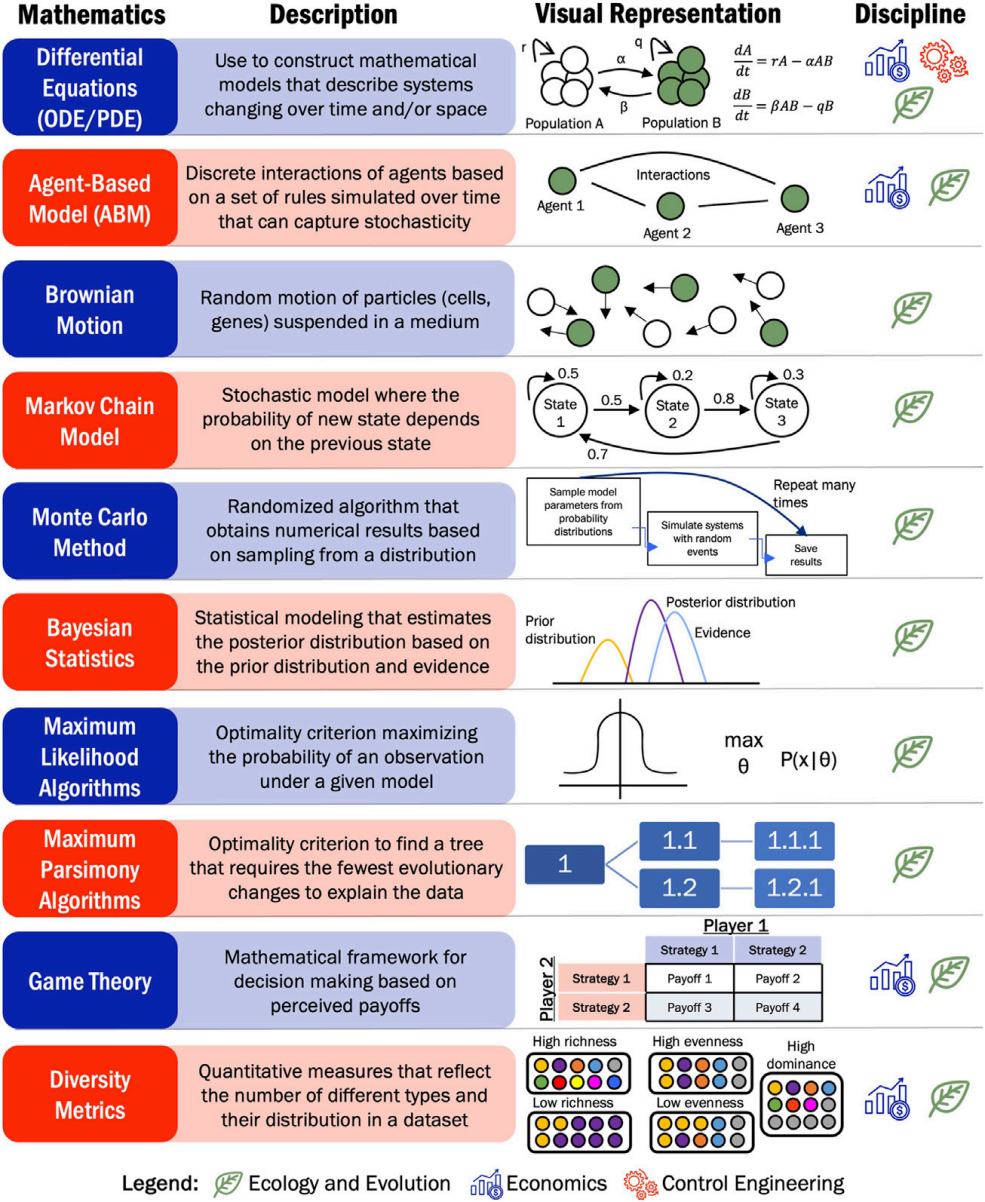
Representation of most commonly applied mathematical frameworks leveraged to answer open questions in cancer research using insights from ecology and evolution, economics and control engineering.

In this review, we have explored how mechanistic mathematical modeling tools from different disciplines can be applied to derive novel insights into progression and treatment response in adenocarcinomas (broadly summarized in Table 1). Being able to study cancer using mathematical concepts (summarized in Figure 3) provides a great advantage when designing treatment strategies, especially in adenocarcinomas that are often asymptomatic until presenting at advanced stages when diagnosed. Computational models able to diagnose or personalize treatment schedules using only a few inputs have the potential to vastly improve patient outcomes and personalize treatment strategies. In this review we have focused on analyzing mechanistic or analytical models that already existed in different fields and were applied to cancer. These models that already describe mechanisms present in different frameworks have been leveraged to obtain novel insights about cancer progression and personalized treatment.

**TABLE 1:** Examples of open questions in cancer research that can be answered using cross-disciplinary insights from ecology and evolution, economics, and control engineering. Broadly these questions can be divided into temporal/dynamical, spatial/environmental, and historical/evolutionary.

Scale	Question	Discipline	Data	Frameworks	Notable applications	Ref.
Temporal/Dynamical	**How** does cancer progress over time?	Ecology & Evolution	Time dependent data (e.g., cell counts, volume changes, biomarker levels … )	ODE, ABM	Lotka-Volterra population dynamics models predicting changes in tumor population size over time	Cerasuolo et al. (2020), Michelson (1987); Michelson et al. (1987); Maley and Forrest (2000); Deisboeck and Wang (2008); Sega and Chignola (2014); Voutouri and Stylianopoulos (2014); Belfatto et al. (2016); Roy and Finley (2017); Zhang et al. (2017); Draghi et al. (2019); Grassberger et al. (2019); Cunningham et al. (2020); Milotti et al. (2020); Reed et al. (2020); Belkhir et al. (2021); Damaghi et al. (2021); Kimmel et al. (2021); Gallaher et al. (2023); Strobl et al. (2023)
	**How** do selective pressures affect it?					
	**How** does cancer progress over time due to random changes?	Ecology & Evolution	Time dependent data (e.g., cell counts, biomarker levels … ), mutation and survival data	ODE, ABM, MCMC models, random walk, functional networks	Models can predict random changes in tumor population size over time	Peirolo and Scalerandi (2004), Veestraeten (2006), Yeh et al. (2008), Hur et al. (2010), Fang and Gough (2014), Curtius et al. (2020), Jiang et al. (2020), Lakatos et al. (2020), López et al. (2020), Curtius et al. (2021), Chen et al. (2022)
	**How** does an individual cell’s strategy affect tumor progression?	Economics	Time dependent data (e.g., cell counts, biomarker levels … ), spatial/location data	Game theory, Trade theory	Game theoretic model describes interacting populations over time based on strategies and pay-offs or trade-offs to explain resistance development, and the emergence of cell states	Kaznatcheev et al. (2015); Malekian et al. (2016); Archetti (2021); Nam et al. (2021); Wölfl et al. (2022), Zhang et al. (2017), Laruelle et al. (2023), Stanková et al. (2019), Hausser and Alon (2020), Boddy et al. (2018)
	**How** can cancer treatment be optimized?	Control Engineering	Time dependent data (e.g., cell counts, fold changes … ), marker expression	Control theory, Decision theory	Optimize cancer systems in real-time until a desired state is reached by identifying optimal treatment schedules	Carrère (2017), Jonsson et al. (2017), Kenn et al. (2021), Plaugher and Murrugarra (2021), Foahom Gouabou et al. (2022)
	**How** do cancer cell populations interact based on resource availability?	Ecology & Evolution	Time dependent data (binding ability), flow cytometry data, ATP competition data	Competition	Identify competitive ability of a population for resources and bind to receptors to produce a downstream effect on cancer progression	Yu et al. (2020), Cheng et al. (2022), Furman et al. (2022)
		Economics				
Spatial/Environmental	**What** is the cellular and molecular diversity of the tumor microenvironment?	Ecology & Evolution	Transcriptomic data (single-cell RNA sequencing data)	Diversity metrics, Gini index	Assess diversity (richness, evenness, dominance) and equity of cell types and distributions in a sample under different conditions	Shannon, 1948; Jost (2006), Crupi et al. (2019), Ferrall-Fairbanks et al. (2019), Hinohara et al. (2019), Filho et al. (2021), Miroshnychenko et al. (2021), Roswell et al. (2021), Ferrall-Fairbanks et al. (2022a), Ferrall-Fairbanks et al. (2022b), Gil Del Alcazar et al. (2022)
	**How** are cells disturbed under different conditions and environments?	Economics				
	**Where** are cells located within the tumor microenvironment?	Ecology & Evolution	Time series volume data, transcriptomic data (bulk and single-cell sequencing data), epigenetic data (DNA methylation sites)	Geospatial measures, ODE/PDE	Provides spatial distribution and clonal evolution patterns of cellular populations to identify malignant clones for treatment	Andres et al. (2001), González-García et al. (2002), Sápi et al., 2015; Yuan (2016), Sievers et al. (2017), Sun et al. (2017), Casasent et al. (2018), Farkas et al. (2018), Dietz et al. (2019), Yamamoto et al. (2019), Efremova et al. (2020), Shan et al. (2022), Karasaki et al. (2023), Yang et al. (2023)
Historical/Evolutionary	**What** biomarkers are responsible for cancer progression?	Ecology & Evolution	Transcriptomic data (single-cell sequencing), genomic data (whole exome sequencing, CNVs and SNVs)	Bayesian, maximum parsimony, tumor clonality, clustering	Retrieve complex phylogenetic topologies in cancer evolution and reveal therapeutic markers	Kostadinov et al. (2013), Leung et al. (2017), Fimereli et al. (2022), Frankell et al. (2023), Turajlic et al. (2015), Nascimento et al. (2017), Toosi et al. (2019), Reiter et al. (2017), Makohon-Moore et al. (2018), Li et al. (2020), Nalbantoglu et al. (2016), Nixon (1999), Murugaesu et al. (2015), von Heydebreck et al. (2003), Petersson et al. (2022), Zhao et al. (2016), V Alekseyenko et al. (2008), McPherson et al. (2016), Wu et al. (2019), Malikic et al. (2015), Abbosh et al. (2017), Prandi et al. (2014), van der Mijn et al. (2022), Niknafs et al. (2015), Niknafs et al. (2019), Saitou and Nei (1987), Siravegna et al. (2018), Matsui et al. (2017), Popic et al. (2015), El-Kebir et al. (2017), Gertz et al. (2016), Roth et al. (2014)
	**What** biomarkers can be therapeutically targeted?					

From ecology, cancer researchers have leveraged frameworks historical applied to describe interacting populations in an ecosystem, to control engineering where cancer researchers have designed and used optimization to regulate systems to meet performance specific tasks, to application of economics metrics to assess wealth and game theoretic interactions, cancer researchers have utilized these foundational concepts to develop a new perspective at understanding tumor dynamics. At the base of all these cross-disciplinary frameworks are the same mathematical modeling approaches including differential equations (ODEs/PDEs), stochastic and probabilistic models, agent-based models (ABMs), Markov models, and Bayesian approaches that have all been aimed to study cancer progression and treatment response. Choosing the most effective cross-disciplinary tool depends on the scientific question and available data. When trying to quantify the diversity of cell types and phenotypes in a tumor, diversity (e.g., Shannon index, GDI) and inequity (Gini index) measures have been used to stratify patients based on disease characteristics and potential treatment response. To learn the location of these cell types across the tumor, spatial ecology concepts could be implemented on transcriptomic or tumor volume data. If the focus is more specific and researchers wanted to identify what biomarker to therapeutically target, then the construction of phylogenetic trees would be optimal. Phylogenetic topologies can provide insights into what biomarker is responsible for cancer progression to aggressive phenotypes.

Most researchers, however, are focused on mechanisms of cancer progression and treatment resistance. How does cancer progress over time? How can cancer be treated effectively? Mathematical concepts have been used to answer these questions. Ecological population-based modeling has provided insights on cancer progression considering interactions between multiple cellular populations. Leveraging concepts such as Lotka-Volterra interactions in mathematical modeling frameworks such as ODEs and ABMs, facilitates the representation of tumor growth as a heterogeneous population with specific interactions between species in the model. Both ODE and ABM frameworks can capture the dynamics tumor growth dynamics, but ABM models can also describe the stochasticity often involved in cancer. Tumors can accumulate mutations over time that may affect their growth or behavior. Thus, to capture randomness associated with cancer progression into differential equation-based models, stochastic and probabilistic frameworks from ecology have been used. These concepts include Markov Chain Monte Carlo (MCMC) approaches, random walk models, or functional networks.

How different cell types affect cancer development is one of the questions that game theory from economics aims to answer. Considering cancer as players able to execute different strategies, game theory explores what are the pay offs of performing one strategy *versus* another. In the context of cancer treatment, the strategies have also been modeled as different treatment options that have different associated payoffs. However, game theory is not able optimize cancer treatment to reach a desired state. Other economic tools such as decision theory could explain how the cell population make decisions based expected outcomes and costs, which could help create precise personalized treatments. While decision theory has mostly been explored in a theoretical realm, researchers have used breast cancer or melanoma data to determine the best decision option (treatment option). This involved identifying the decision/treatment that maximizes a desired outcome, as determined by the decision maker (Foahom Gouabou et al., 2022; Kenn et al., 2021).

Similarly, concepts from economics such as trade theory or competition assays, that were originally used to assess the exchange of goods or competitive fitness, respectively, can be applied to cancer. Trade theory has conceptually been used to identify cellular trade-offs when performing different biological tasks (Hausser and Alon, 2020; Boddy et al., 2018). On the other hand, competition assays have focused on studying the ability of different molecules to bind to a receptor and produce a downstream effect on cancer progression (Yu et al., 2020; Cheng et al., 2022; Furman et al., 2022). Contrary to Lotka-Volterra interactions that assess population dynamics, economic competition assays focus on resource competition and cellular interactions based on the exchange of signals. Based on the constraints of the literature search utilized here, the manuscripts identified with mathematical modeling of economics concepts applied to adenocarcinomas were confined to EGT and the Gini index. Trade theory, competition assays, and decision theory are additional concepts from economics that represent an opportunity for researchers to further utilize this cross-disciplinary space to continue to identify novel applications of these principles in cancer research. Control engineering uses a similar approach to decision theory where cancer progression is optimized to a final state. Modeling cancer as a control system provides the opportunity to design personalized treatment schedules to achieve a desired outcome, such as minimizing tumor growth or preventing the development of resistance.

Intuitively, the selection of a modeling approach depends on the biological question, data availability, and computational resources. Additionally, there are several limitations that all these frameworks encounter when analyzing or predicting tumor dynamics. Populations models consider interactions among a few cell populations and physiological agents. However, the entire tumor microenvironment involves additional factors like immune or epithelial cells, and physiological conditions such as pH, oxygen, or glucose levels. These elements influence cancer growth differently depending on the tumor structure and arrangement of cells, which are not addressed in the 2D tumor models presented in this review. Moreover, most cancer progression models presented here are deterministic and fail to capture stochastic events characteristic of cancer progression. While phylogenetic algorithms do a great a job at recreating possible evolutionary cancer trajectories by accounting for randomness, using the wrong algorithm for a certain data type could lead to incorrect trajectories and indicators for treatments. Another limitation, and opportunity for future work, in using these approaches is that they are not yet robust to leverage the ever expanding different types of input data available in cancer research. Some cancer biomarker data becoming more popular that could be combined with the approaches detailed here include circulating tumor DNA (ctDNA) and cell-free DNA (cfDNA). In these approaches, DNA fragments shed from the tumor into the blood stream and can be collected using liquid-biopsies (Dao et al., 2023). While using this data offers great opportunities to monitor disease status and treatment response, the utilization in mechanistic models has been lacking and most approaches rely on non-analytical models to interpret their significance. Consequently, more work needs to be done to effectively use information obtained from these clinically useful measures to enhance the potential predictive power and utility of the mechanistic modeling approaches presented in this review. Additionally, the refinement of these mechanistic approaches provides an opportunity to study other cancer types, such as childhood cancers, that present different cancer progression mechanisms.

Utilizing the foundational concepts from ecology, economics, and control theory has provided useful information to predict tumor response and progression under different environmental conditions. However, these frameworks have largely been applied to small cohorts of *in vivo* and *in vitro* environments, which fail to capture the overall picture of the disease. Clinically, most cancers are treated using maximum tolerated doses of chemotherapeutics which leads to resistance development and high toxicities on the host. Understanding how each patient’s disease develops throughout the treatment course is a crucial step when developing these models. Therefore, a clinician’s perspective is required when developing translational mathematical models of tumor progression. In addition to tumor size changes across different timepoints during treatment, a patient’s previous history can be used when developing these models making them patient specific. Once a model is adjusted for each patient, different treatment strategies can be explored virtually, and modelers and oncologists would be able to decide the most optimal treatment. After treatment, the model can be refined based on the patient’s response. Collaborations between clinicians and modelers must be develop for these strategies to be successful. Some institutions are starting to have success in this area as demonstrated with the idea of an Evolutionary Tumor Board for personalizing treatment strategies (Robertson-Tessi et al., 2023). Modelers could also request additional patient data such as sequencing information or biomarker expression to inform the design of their model. Bringing clinicians and additional patient data can improve patient prognosis. However, challenges arise from the lack of previous patient medical history, insufficient equipment to collect necessary tumor characteristics, and uncertainty when selecting a modeling tool or framework. To overcome these challenges, researchers have started exploring diverse modeling strategies to available datasets to predict cancer evolution in cancers with different characteristics. As *in silico* mathematical tools become more widely used, it is crucial for clinicians and other experts to collaborate closely with cancer researchers. These collaborations will help identify and address any additional limitations that may arise.
